# A Low-Power Complementary Metal-Oxide-Semiconductor Receiver with Quadrature Bandpass Continuous-Time Delta–Sigma Analog-to-Digital Converter for IoT Applications

**DOI:** 10.3390/s25061748

**Published:** 2025-03-12

**Authors:** Nam-Seog Kim

**Affiliations:** Department of Information and Communication Engineering, School of Electrical and Computer Engineering, Chungbuk National University, Cheongju-si 28644, Republic of Korea; namseog.kim@cbnu.ac.kr

**Keywords:** Bluetooth Low Energy (BLE), continuous-time delta–sigma ADC, complex bandpass ADC, CMOS, Internet of Things (IoT), low-power receiver, wireless sensor networks (WSN), low-IF architecture, image rejection, energy efficiency

## Abstract

This paper presents a low-power CMOS receiver with a complex continuous-time delta–sigma ADC designed for IoT applications in the 2.4 GHz band. The architecture employs a quadrature bandpass continuous-time delta–sigma ADC optimized for Bluetooth Low Energy (BLE) standards, achieving an ENOB of 10.9 bits while consuming only 0.81 mW from a 1.0 V supply. The receiver demonstrates impressive performance metrics, including a sensitivity of −95 dBm at a 10⁻^3^-bit error rate, an image rejection ratio of 54.2 dBc, and a spurious-free dynamic range of 79.8 dBc. Operating at a 1.5 MHz intermediate frequency with a 2 MHz bandwidth, the ADC achieves superior energy efficiency with a figure of merit (FOM_W_) of 103.2 fJ/conv. Implemented in 28 nm CMOS technology, the complete receiver occupies 0.375 mm^2^ for the RF front-end and 0.145 mm^2^ for the ADC while consuming 4.08 mW total power, making it well suited for battery-powered IoT sensor nodes requiring both power efficiency and reliable wireless connectivity.

## 1. Introduction

The Internet of Things (IoT) has emerged as a transformative paradigm, revolutionizing how we interact with our environment. As IoT devices grow exponentially, there is increasing demand for low-power, cost-effective wireless communication solutions [1,2]. This proliferation of interconnected smart devices necessitates innovative receiver designs that meet stringent IoT requirements while maintaining energy efficiency and compact form factors.

In IoT receiver design, the low-IF (intermediate frequency) architecture has gained traction by balancing power efficiency and performance. This architecture offers advantages over traditional direct conversion (zero-IF) receivers, including improved DC offset and flicker noise performance. However, low-IF faces image rejection challenges crucial for maintaining signal integrity amid interference. To address this, complex continuous-time sigma–delta analog-to-digital converters (ADCs) provide an effective solution. These ADCs perform both analog-to-digital conversion and image rejection, simplifying receiver architecture while maintaining high performance. Their continuous-time implementation provides inherent antialiasing filtering, reducing analog filtering requirements before the ADC stage.

**Figure 1:**
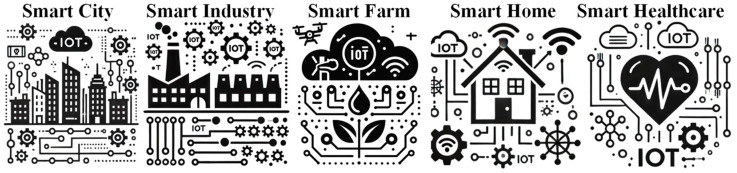
IoT applications utilizing low-power CMOS receivers in the 2.4 GHz ISM band.

The 2.4 GHz ISM (Industrial, Scientific, and Medical) band has become popular for IoT applications due to its global availability and support for protocols like Bluetooth Low Energy (BLE) [2] and Zigbee [3], as shown in Figure 1. These protocols suit short-range communication and offer low power consumption, making them ideal for battery-powered IoT devices.

IoT receiver design faces several critical challenges. Power consumption is paramount, as IoT devices must operate long-term on battery power or energy harvesting. This demands ultra-low-power circuits and architectures that maintain performance while minimizing energy use. The evolving IoT ecosystem requires flexible receivers supporting multiple communication protocols, with adaptable modulation schemes and data rates at low power consumption.

Integration presents another challenge. Single-chip solutions must incorporate complex functionalities while reducing system cost and size for space-constrained IoT devices. This necessitates innovative circuit topologies and system architectures that achieve high integration without performance compromise. The analog-to-digital converter design significantly impacts overall receiver performance [4,5]. ADCs must handle wide dynamic ranges and maintain resolution across varied signal conditions while consuming minimal power. Continuous-time sigma–delta ADCs offer an effective balance between performance and power efficiency for these requirements [6].

This paper presents a low-power CMOS receiver for 2.4 GHz ISM band IoT applications, compatible with the BLE protocol. The design combines low-IF architecture with a complex continuous-time sigma–delta ADC for efficient image rejection and high-performance analog-to-digital conversion. Our integrated approach creates a power-efficient, compact receiver that meets modern IoT requirements. The design emphasizes minimal power consumption while maintaining BLE performance standards. The complex continuous-time sigma–delta ADC serves dual purposes: bandpass filtering for image rejection in low-IF receivers and simplified architecture for reduced power consumption and improved signal quality.

The subsequent sections provide a detailed examination of the system architecture, focusing on three key aspects: First, we present the design considerations for implementing a quadrature bandpass continuous-time delta–sigma ADC optimized for BLE applications. Second, we analyze the circuit-level implementations, including the 4-bit feedback DAC, active-RC integrator, and SAR-based quantizer designs. Finally, we evaluate the system’s performance through comprehensive measurements of the signal-to-noise ratio (SNR), signal-to-noise-and-distortion ratio (SNDR), image rejection ratio (IRR), and power consumption. The proposed architecture incorporates several innovative techniques, such as a thermometer-coded capacitor array layout for improved matching, DWA-based linearization for the feedback DAC, and an RC calibration scheme that maintains accurate frequency response across process, voltage, and temperature (PVT) variations. These optimizations collectively result in a power-efficient receiver achieving −95 dBm sensitivity and 54.2 dBc image rejection while consuming only 4.08 mW, making it particularly suitable for battery-operated IoT sensor nodes in the 2.4 GHz band.

## 2. A Comparative Analysis of IoT Receiver Architectures

**Figure 2:**
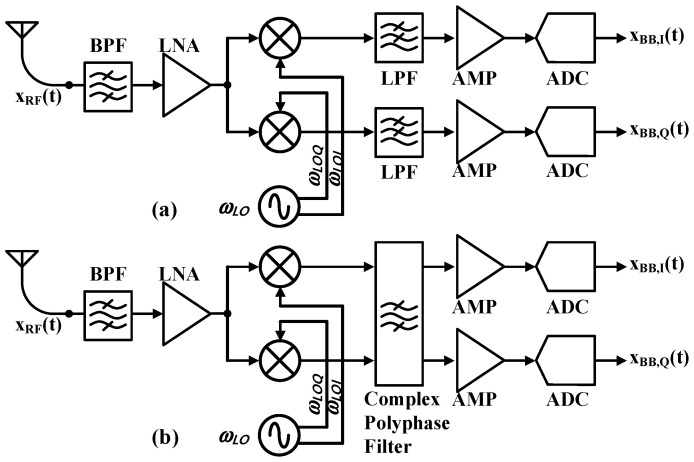
Block diagram of (**a**) direct-conversion and (**b**) low-IF receivers.

Direct conversion and low-IF represent the primary receiver architectures for BLE protocol IoT applications, as shown in Figure 2 [7]. Direct conversion receivers down-convert RF signals to baseband, offering a simpler design and lower power consumption, but suffer from DC offset and flicker noise issues [8]. Low-IF receivers convert to a non-zero intermediate frequency, providing better immunity to these issues at the cost of higher power consumption and complexity [9].

For 2.4 GHz ISM band BLE applications, architecture choice significantly impacts system performance. Direct conversion receivers particularly struggle with I/Q mismatch and DC offset issues, affecting packet reception rates [10].

Low-IF receivers provide superior DC offset immunity through non-zero intermediate frequency processing—crucial for IoT applications where power constraints limit DC offset compensation [11]. This architecture inherently guards against flicker noise, essential for narrow-band BLE protocols with DC-concentrated baseband signals [12].

The low-intermediate-frequency processing enables effective channel filtering and adjacent channel rejection, improving selectivity in dense IoT deployments [9]. Low-IF better handles I/Q imbalance versus direct conversion, enhancing demodulation reliability for complex modulation schemes [13]. Additionally, the absence of DC offset simplifies automatic gain control implementation, improving dynamic range handling [14]. However, to avoid corrupting the desired signal, the image signal needs to be suppressed. While complex bandpass filters are effective for image rejection in low-IF receivers [15], they often lead to increased power consumption [16]. The integration of bandpass quadrature sigma–delta modulators presents a promising solution to reduce this power demand while maintaining performance.

## 3. Optimizing ADC Dynamic Range for BLE Protocol: A Comprehensive Analysis

**Figure 3:**
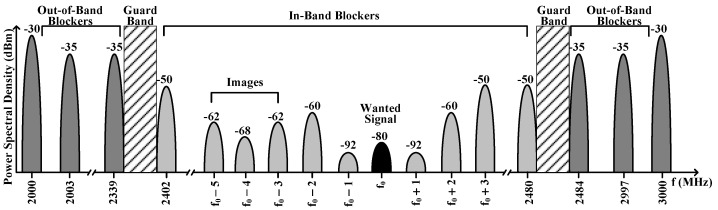
Blocker mask for the BLE Standard.

BLE receiver ADC specifications demand precise balancing to achieve optimal system performance. The ADC must deliver a dynamic range exceeding 60 dB to effectively process signals ranging from weak desired signals at −77 dBm to strong adjacent channel blockers at −50 dBm, as illustrated in Figure 3 [17].

The system incorporates essential margin requirements for reliable operation. A 10 dB back-off margin prevents the ADC’s inherent noise floor from degrading receiver sensitivity, while an additional 6 dB margin accommodates the stepped transitions of automatic gain control (AGC) implementations. These combined margins ensure stable system operation across varying signal conditions. The ADC must maintain a minimum SNR of 9 dB even under maximum blocker conditions, translating to approximately 4-bit resolution when operating at sampling frequencies between 10 and 14 MHz. This specification balances quantization noise requirements with processing bandwidth (BW) constraints.

An SNDR target of 24 dB has proven sufficient for maintaining robust system performance. The ADC’s performance integrates closely with the baseband filtering architecture, where digital complex bandpass filtering provides crucial antialiasing and image rejection functions. This filtering maintains a bit error rate (BER) of 0.1% or better by attenuating out-of-band blockers before they can saturate the ADC’s input stage. Performance enhancement through oversampling techniques offers additional resolution improvements.

Implementation requirements demand that the ADC handle the complete range of signal conditions specified in the BLE standard [18]. The sampling frequency must be carefully selected to capture the required signal BW while preventing interferer aliasing. These comprehensive specifications ensure effective BLE signal digitization with robust performance amid various interferers and blockers in the increasingly congested 2.4 GHz ISM band. The successful integration of resolution, sampling rate, and filtering requirements enables reliable wireless communication in this challenging environment.

## 4. Complex Continuous-Time Delta–Sigma ADCs: Principles and Challenges

**Figure 4:**
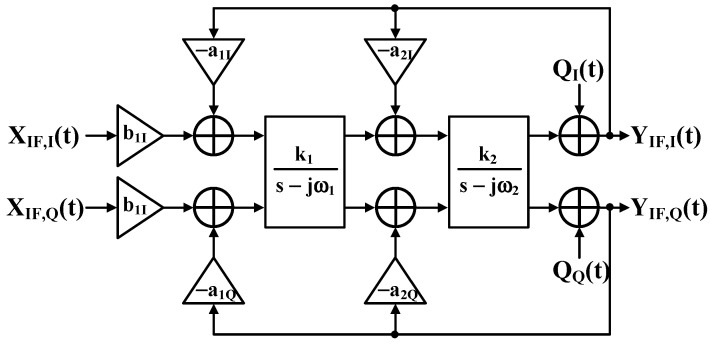
Block diagram of complex bandpass continuous-time delta–sigma modulator.

Complex bandpass continuous-time sigma–delta analog-to-digital converters (CTΔΣADCs) offer sophisticated bandpass signal digitization, as shown in Figure 4 [19]. These ADCs excel in software-defined radio and low-IF receiver applications by mitigating image problems without complex analog image-reject filters. The architecture uses a feedback-type quadrature bandpass continuous-time sigma–delta modulator (QB-CTΔΣM), implementing complex integrators through cross-coupled I/Q integrator pairs [20].

The feedback topology delivers key advantages in the signal transfer function (STF): superior filtering characteristics, enhanced antialiasing without gain peaking, and elimination of the feedforward designs’ summing stage before quantization [21]. This architecture effectively handles out-of-band blockers, which often exceed the desired in-band signal strength in receiver applications [22].

The sigma–delta loop’s complex signal processing implements optimized transfer functions through careful noise transfer function design and strategic complex notch placement at the signal band center frequency [23]. This results in doubled noise-shaping capability over conventional real bandpass modulators.

**Figure 5:**
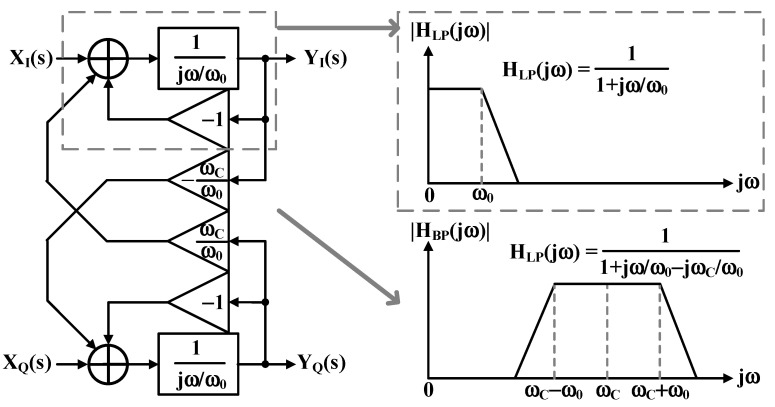
Block diagram and transfer functions H (jω) of QBP-CTΣΔM.

Each QB-CTΣΔM stage comprises two cross-coupled low-pass CTΣΔM (LP-CTΣΔM) units. Figure 5 illustrates the block diagram of a single QB-CTΣΔM stage [24]. The single-loop LP-CTΣΔM filter transfer function H_LP_(jω) is expressed in Equation (1) and depicted by the dotted line in Figure 5.

(1) $$H_{\mathrm{LP}}\left(j\omega \right)=\frac{1}{1+j\omega /\omega_{0}}$$

The single-loop QB-CTΣΔM transfer function H_BP_ (jw) is derived by the frequency shifting from H_LP_(jω) to center frequency ω_C_, substituting s-jωC for s. This shifts the LP-CTΣΔM cutoff frequency ω_0_ to ω_C_ ± ω_0_. Equation (2) describes the resulting H_BP_(jw), with Q representing the QB-CTΣΔM quality factor.

(2) $$H_{\mathrm{BP}}\left(j\omega \right)=H_{\mathrm{LP}}\left(j\omega -j\omega_{C}\right)=\frac{1}{1+j\omega /\omega_{0}-j\omega_{C}/\omega_{0}}$$

The implementation of an ADC with QB-CTΣΔMs presents several critical challenges that must be carefully addressed. Path mismatches between I/Q channels can significantly degrade image rejection performance, necessitating careful layout techniques and, potentially, calibration mechanisms [25]. Clock jitter sensitivity is another crucial concern, as timing errors in the feedback DAC directly impact conversion accuracy, requiring robust clock distribution networks and carefully designed DAC switching schemes [26]. The feedback topology, which must process both the input signal and noise, demands appropriate filter coefficient selection and circuit topology optimization to accommodate larger signal swings effectively. Stability considerations become particularly complex due to interactions between I/Q paths, requiring comprehensive stability analysis that accounts for variations in operating conditions [27]. Additionally, various non-idealities—such as I/Q mismatches, DAC element mismatching, and excess loop delay—often necessitate sophisticated calibration schemes [28].

Despite these challenges, the continuous-time implementation offers several advantages over discrete-time alternatives, including inherent antialiasing filtering, lower power consumption, higher potential sampling rates, and freedom from switched-capacitor settling constraints [29]. These benefits make complex CT-ΣΔADCs particularly well suited for modern wireless communication systems, especially in IoT applications, where power efficiency and performance must be carefully balanced.

## 5. Proposed Quadrature Bandpass Continuous-Time Delta–Sigma ADC

### 5.1. Quadrature Bandpass Continuous-Time Delta–Sigma ADC Architecture

**Figure 6:**
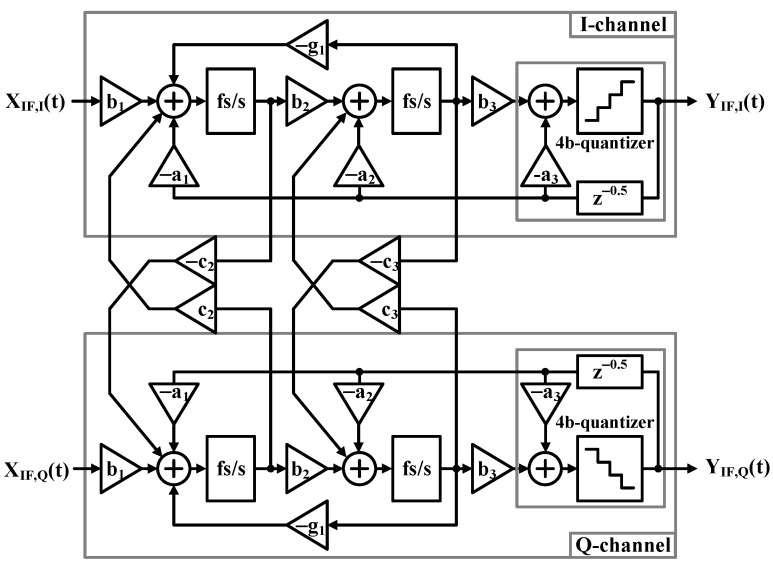
Block diagram and transfer functions H (jω) of QBP-CTΣΔM.

The proposed receiver architecture incorporates a quadrature bandpass continuous-time delta–sigma analog-to-digital converter (CT-ΣΔ ADC) specifically designed to meet BLE specifications, as shown in Figure 6. The ADC implementation features carefully selected performance parameters that align with the requirements of modern wireless communication systems, with a particular focus on BLE applications. Within its operational specifications, the ADC demonstrates a robust in-band dynamic range, which is crucial for reliable wireless communication.

The system processes signals within an integrated BW of 2 MHz, operating in the frequency range of 0.5 MHz to 2.5 MHz. This range is strategically chosen to optimize receiver performance while maintaining compatibility with BLE signal requirements.

In terms of signal handling, the ADC achieves a dynamic range of 60 dB, enabling it to process both strong and weak signals effectively. This specification ensures the receiver can accommodate the wide variation in signal strengths typically encountered in BLE applications, from nearby devices to those at the maximum specified range.

The architecture’s continuous-time implementation provides inherent antialiasing capabilities, achieving over 39 dB attenuation at the first sampling zone. This built-in antialiasing functionality is a significant advantage of the continuous-time approach, effectively suppressing unwanted high-frequency components that could otherwise interfere with the desired signal.

Furthermore, the system exhibits strong image rejection capabilities, with an image rejection ratio specified to be better than −32 dBc. This specification ensures that the receiver can effectively suppress signals at the image frequency, which is crucial for maintaining signal quality and preventing interference in the quadrature receiver architecture.

These comprehensive specifications demonstrate that the ADC design meets the necessary performance metrics to support reliable BLE communication while maintaining practical implementation considerations. The combination of BW, dynamic range, antialiasing, and image rejection capabilities makes this architecture particularly well suited for modern wireless communication applications, especially within the context of BLE systems.

### 5.2. Quadrature Bandpass Continuous-Time Delta–Sigma ADC Circuit Design

#### 5.2.1. Four-Bit Feedback Current Digital-to-Analog Converter Circuit

**Figure 7:**
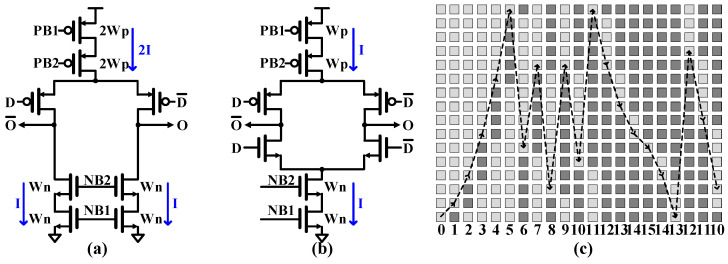
(**a**) Unipolar and (**b**) bipolar current DAC cells and (**c**) 4-bit DWA pointer rotation.

Four-bit feedback digital-to-analog converters (FBDACs) are circuits that implement the negative feedback factors a_1_, a_2_, a_3_, and g_1_ of the QBP-CTΣΔM, as shown in Figure 6. They employ the bipolar topology depicted in Figure 7b rather than the unipolar approach shown in Figure 7a, which allows the DAC current to be reduced by half compared to the unipolar version. To improve the output impedance of the DAC unit cell, cascode transistors are used in the current source, utilizing both NMOS and PMOS configurations. In the current design, the unit current of the bipolar topology is specifically designed to be half that of the unipolar topology. This design choice serves two purposes: reducing noise contribution and lowering current consumption in the bipolar topology. Additionally, it enables smaller capacitor loads, resulting in a more compact overall design.

The linearity of the DAC is critical for the QBP-CTΣΔM to achieve the required SNR. To address DAC linearity issues, a data-weighted averaging (DWA) technique is implemented, effectively mitigating these concerns without requiring large device areas for intrinsic device size matching. The DWA system operates by updating the pointer location in each cycle based on the current position and incoming data, as shown in Figure 7c.

The pointer rotation process begins with the initialization of a pointer, typically set to zero, which serves as the starting point for element selection. When a digital input sample enters the DAC, it undergoes conversion into a thermometer code, representing the number of elements that need to be activated for that particular input. The DWA algorithm then proceeds to select DAC elements sequentially, starting from the current pointer position. It activates as many elements as required by the input code, ensuring that each element contributes equally over time. After processing the input sample, the pointer is updated to indicate the next unused element, preparing the system for the subsequent input. This continuous update mechanism is crucial for maintaining the balanced usage of all DAC elements. In cases where the pointer reaches the end of the available DAC elements, it employs a wraparound technique, returning to the beginning of the element array. This wraparound ensures that the rotation process continues seamlessly, maintaining the equal utilization of all elements over extended periods of operation. By consistently rotating through all available elements, the DWA pointer rotation process effectively distributes any mismatch errors across the entire DAC, resulting in improved overall linearity and performance. Notably, the pointer-shifting logic for the DWA is kept separate from the main feedback path, ensuring that the pointer for the current data is immediately available without introducing additional logic delays. As a result, the only delay in the system comes from data propagation through the matrix.

**Figure 8:**
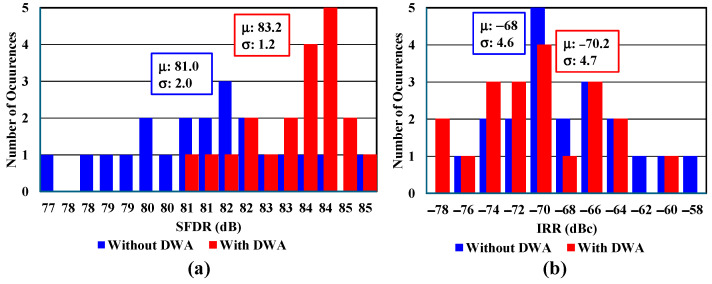
Twenty Monte Carlo post-simulations without DWA and DWA for (**a**) SFDR and (**b**) IRR.

Figure 8 shows Monte Carlo simulation results comparing the performance of a complex bandpass sigma–delta ADC with and without DWA. The results are presented in two histograms: one for spurious-free dynamic range (SFDR) and one for IRR.

In the SFDR analysis, the two configurations show distinct characteristics. Without DWA, the system achieves a mean of −75.1 dB with a standard deviation of 1.1 dB. When DWA is implemented, the performance improves to a mean of 83.2 dB with a standard deviation of 1.2 dB, demonstrating significant enhancement in spurious performance. The distribution of SFDR values spans from approximately 72 dB to 85 dB, showing consistent performance across multiple simulation runs.

For the IRR measurements, there is a notable difference between the two configurations. Without DWA, the system achieves a mean IRR of −68 dB with a standard deviation of 4.6 dB. When DWA is implemented, the mean IRR slightly decreases to −70.2 dB with a standard deviation of 4.7 dB, indicating a minor degradation in image rejection performance. The IRR values range from approximately −78 dBc to −58 dBc across all simulations.

The timing structure of the system is carefully designed, with the feedback DAC’s final injection occurring at 0.5 T after the quantizer’s sampling edge. This timing allocation ensures sufficient processing time for three critical operations: SAR ADC subtraction, conversion, and DWA data transmission. Performance testing through 20 Monte Carlo post-simulations reveals impressive overall results, with the system achieving an SFDR greater than 80 dB and a signal-to-quantization-noise ratio (SQNR) exceeding 77 dB. The worst-case IRR of approximately 60 dBc is deemed sufficient to make the ADC’s contribution to image rejection negligible in the overall system performance.

**Figure 9:**
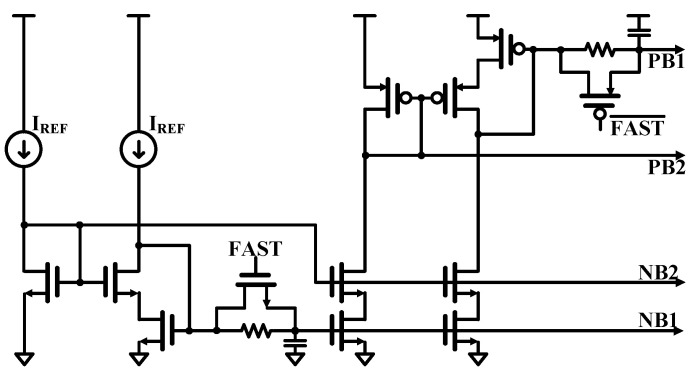
FBDAC bias circuit.

In the bias circuit design shown in Figure 9, the system operates with a 1 V supply voltage provided by an internal regulator, supporting a mismatch range of approximately ±10%, from 0.9 V to 1.1 V. Due to limited voltage headroom, source degeneration resistors are excluded from the design. The low-IF receiver design minimizes sensitivity to flicker noise, with thermal noise from the devices being the predominant noise source. To achieve the required dynamic range of over 60 dB, the transistors are designed with increased length and decreased width, resulting in lower transconductance (g_m_) and higher saturation voltage (V_DSAT_).

Noise from the bandgap and bias generator circuits degrades dynamic range due to the receiver’s 2 MHz wide BW; the bias circuit implements an RC filter with a cutoff frequency of 48 kHz. To mitigate potential long settlement times caused by the RC filter, the design includes a fast-settle shorting switch activated by a loop filter reset signal. This approach achieves a settlement time of less than 30 μs, including the bandgap, bias generator, and loop filter reset timing, meeting the BLE system specification of 90 μs.

#### 5.2.2. Active-RC Integrator with Proposed OTA

**Figure 10:**
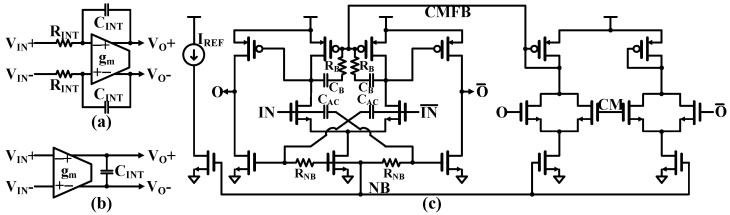
(**a**) Active-RC and (**b**) Gm-C integrators and (**c**) transconductance amplifier (OTA).

Continuous-time loop filters can be implemented using either active-RC or Gm-C integrators, as shown in Figure 10a,b, each with distinct characteristics and trade-offs. For the proposed QBP-CTΣΔM, active-RC integrators are the preferred implementation approach, as they deliver enhanced linearity at comparable power consumption levels and can handle larger signal swings compared to Gm-C integrators. Additionally, operational transconductance amplifiers (OTAs) create more stable virtual grounds through closed-loop operation, independent of input conditions. While Gm-C integrators excel in terms of power efficiency and can achieve higher operational speeds, active-RC integrators are the more suitable choice for high-speed CTDSM due to their higher maximum stable input amplitude.

A two-stage amplifier can provide a higher overall gain compared to a single-stage amplifier. This is crucial for active-RC integrators, as it enhances accuracy and linearity in the integration process. However, two-stage amplifiers often employ Miller compensation to stabilize the circuit and prevent oscillations. Miller compensation typically requires a larger compensation capacitor, leading to increased power consumption because more power is needed to charge and discharge the larger capacitor.

To address this, a fully differential OTA without Miller compensation is employed in this work, as shown in Figure 10c. The OTA utilizes conventional differential difference amplifier-based common-mode feedback (CMFB). During DC operation, the boosting capacitors (C_B_) act as open circuits, while the bias resistors (R_B_) and common-mode feedback (CMFB) circuit establish proper biasing conditions for the fully differential amplifier. In AC operation, the boosting capacitors C_B_ cause the load transistors of the first stage to operate as diode-connected loads at high frequencies. This diode-connected operation reduces the output impedance of the first stage, pushing its pole frequency higher than the second stage’s output pole frequency. With the first-stage pole at a higher frequency than the second-stage pole, Miller compensation becomes unnecessary. Moreover, the input signals are uniquely connected to the second stage through AC coupling capacitors C_AC_. This configuration enables higher gain by bypassing the first stage’s gain limitations. Additionally, the cross-coupled structure in the second stage further enhances the overall gain of the amplifier. It achieves a unity-gain BW of 200 MHz and a noise level of <30 nV/√Hz while consuming 70 µW.

RC calibration of the active-RC integrator plays a crucial role in the QBP-CTΣΔM, as it determines the frequency characteristics of the overall signal through resistance and capacitance values [30]. These values are significantly affected by PVT variations. Both integrator transconductance [31] and capacitance tuning were commonly implemented [32] in gm-C integrators. To compensate for variations in an active-RC integrator, the system employs an active-RC oscillator that integrates resistors, capacitors, and operational transconductance amplifiers (OTAs) in a straightforward switching configuration. The calibration process relies on an external reference frequency of 32 MHz (f_REF_), which remains relatively stable under PVT variations.

The calibration methodology operates by comparing the ratio of reference clock cycles (N) to internal oscillator cycles (K), expressed mathematically as Equation (3).

(3) $$\frac{f_{\mathrm{REF}}}{N}=\frac{f_{\mathrm{OSC}}}{K}$$

To ensure accurate detection of capacitor variations, the system requires that N must be greater than or equal to 1, specifically to detect changes in capacitor LSB value corresponding to 2% capacitive variation, following Equation (4).

(4) $$\frac{K\cdot \left(0.02\cdot f_{\mathrm{REF}}\right)}{f_{\mathrm{OSC}}}>1$$

The capacitor bank is designed with both extensive range and precise resolution capabilities. It spans from 1.1 pF to 4.4 pF, utilizing 1.1 pF as the basic unit and incorporating a 25 fF LSB. This configuration allows for capacitance adjustments between 732 fF and 1.766 pF. The system implements 5-bit controls to accommodate all PVT conditions, including resistance variations, with a fixed resistor value of 32 kΩ. The RC product is carefully configured to achieve approximately 2% resolution, allowing for an acceptable error margin of ±1%.

The calibration process utilizes a Successive Approximation Register (SAR) method, targeting an oscillator frequency (f_OSC_) of 2 MHz. During calibration, the system counts reference clock cycles over four cycles of the active-RC oscillator to achieve ±1% accuracy under ideal conditions. This count is then compared against a predefined reference value of 128. To ensure precise counting, the counter reset is synchronized with each clock cycle. Upon completion and verification of the final bit, the system generates an end-of-conversion signal, and the calibration values are stored in eFuse as serial peripheral interface values for future calibrations. The system demonstrates performance across all PVT corners, achieving frequency convergence to 2 MHz with an accuracy between 98.9% and 104% of the target frequency. The capacitor codes demonstrate appropriate variation across different process corners and temperatures, reflecting the system’s adaptability. The codes range from 31 or 30 at the lowest corner (FF process, highest voltage, and coldest temperature) to 1 or 0 at the highest corner (SS process, lowest voltage, and hottest temperatures). This range utilizes the full span of the 5-bit control for the capacitor bank, which allows for 32 possible settings (0 to 31). Throughout this range, the system consistently maintains the target frequency within the specified accuracy range. This comprehensive calibration approach effectively addresses the challenge of achieving linear coverage across the entire range of PVT variations while maintaining the desired resolution in the active-RC integrator.

#### 5.2.3. Four-Bit Quantizer

A delta modulator quantizes the difference between the current input data and the previous output data with a specific feedback gain, which is accomplished by an SAR-based quantizer through analog subtraction during the sampling phase. Conventional SAR-based quantizers are limited to unity feedback gain, as they share reference voltages between the sampling and conversion phases. Implementing non-unity feedback traditionally requires additional voltage references, which increases circuit complexity, area requirements, and static power consumption [33].

The proposed SAR-based quantizer design implements non-unity feedback gain through a capacitive digital-to-analog converter (CDAC) approach. This design achieves its functionality while maintaining a simple architecture and low power consumption by modifying the ratio of two CDACs during the sampling and conversion phases.

**Figure 11:**
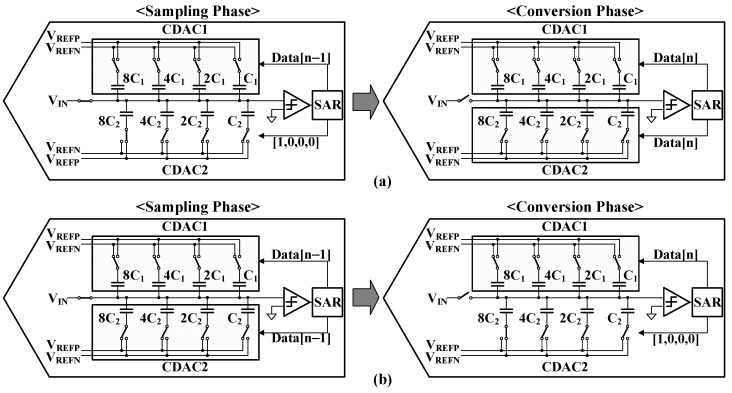
SAR-based quantizer operation for feedback gains of (**a**) <1 and (**b**) >1.

The system’s operation is divided into two distinct phases: sampling and conversion. During the sampling phase, the behavior of the circuit varies depending on the desired feedback gain (α). When the feedback gain needs to be less than unity, the CDAC used in the sampling phase is designed to be smaller than the one used in the conversion phase, as illustrated in Figure 11a. In this configuration, the feedback gain is calculated using Equation (5).

(5) $$\alpha =\frac{CDAC1}{CDAC1+CDAC2},$$

which ensures the gain remains less than one. Conversely, when a feedback gain greater than unity is required, the sampling phase CDAC is designed to be larger than the conversion phase CDAC, as shown in Figure 11b. For this configuration, the feedback gain is calculated using Equation (6).

(6) $$\alpha =\frac{CDAC1+CDAC2}{CDAC1}\;\;\;\;,$$

which yields a value greater than one. Both CDACs utilize binary-weighted capacitor arrays with values of 8C, 4C, 2C, and C to achieve precise digital-to-analog conversion.

A crucial aspect of the design is the handling of reference voltages. The positive reference voltage (V_REFP_) must be scaled to α times larger than that of a conventional SAR-based quantizer to maintain consistent quantization step size. This scaling is accomplished through an adjustable voltage reference generator, which works in conjunction with the CDAC structure.

The overall architecture demonstrates an elegant solution to achieving variable feedback gains without requiring complex analog circuitry. By utilizing capacitor ratio modifications and an adjustable reference voltage generator, the design maintains high performance while keeping power consumption low. The binary-weighted capacitor arrays in both CDACs ensure precise conversion, while the flexible feedback gain adjustment through CDAC ratios provides versatility in application.

This approach to implementing non-unity feedback gain represents a significant advancement in SAR-based quantizer design, offering a power-efficient solution that maintains performance without sacrificing simplicity. The design’s ability to accommodate both greater-than-unity and less-than-unity feedback gains through the same basic architecture demonstrates its versatility and efficiency.

**Figure 12:**
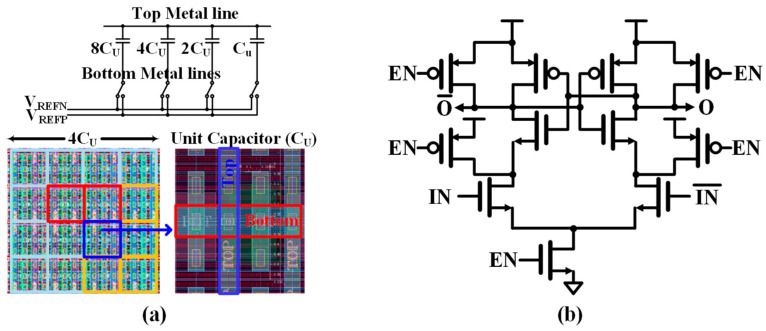
(**a**) Layout view of capacitor array of CDAC and (**b**) StrongARM comparator.

The layout in Figure 12a shows a binary-weighted capacitor array design for the CDAC that incorporates an important design principle: the use of identical unit capacitors (C_U_) as building blocks. Unlike traditional binary-weighted implementations that vary the physical capacitor sizes, this design achieves binary weighting through parallel combinations of identical unit capacitors. Specifically, segments are created by connecting multiple identical C_U_s in parallel to achieve binary scaling (8C_U_, 4C_U_, 2C_U_, and C_U_). This implementation approach preserves the area efficiency of binary weighting while borrowing the matching advantages typically associated with thermometer coding. By constructing all capacitive elements from identical C_U_s rather than physically scaling capacitor dimensions, the layout achieves better matching and improved linearity. This technique minimizes the effects of process variations since all capacitive elements use identical building blocks, providing similar matching advantages as seen in thermometer-coded designs while maintaining binary-weighted functionality.

The layout employs a sophisticated common-centroid technique where these identical unit capacitors are distributed symmetrically around a central point. This arrangement helps cancel out systematic process gradients and temperature variations across the array. The common-centroid pattern is clearly visible in the zoomed-in view of the unit capacitor structure, showing the careful placement and orientation of the identical elements. The structure features a top metal line serving as a common connection point, while individual bottom metal lines connect to the binary-weighted segments composed of the unit capacitors. The reference voltages V_REFN_ and V_REFP_ are routed through switches to each segment, enabling the binary-weighted switching operation during the conversion process.

The StrongARM comparator is shown in Figure 12b [34]. When the clock is low, all internal nodes are precharged to V_DD_ through reset transistors, which minimizes memory effects. When the clock transitions to a high state, the evaluation phase begins as the tail current transistor activates and the reset transistors turn off. This allows the differential input pair to create an initial voltage imbalance at the internal nodes. This imbalance is then amplified by cross-coupled PMOS and NMOS pairs through positive feedback, which drives the outputs rapidly to opposite rails based on the input voltage difference. The StrongARM comparator achieves high speed and energy efficiency due to this regenerative positive feedback mechanism. This characteristic makes it particularly suitable for high-speed ADC applications where fast decision-making is crucial. The design of the comparator requires careful consideration of transistor sizing and layout symmetry to minimize offset and ensure reliable operation across variations in process, voltage, and temperature.

**Figure 13:**
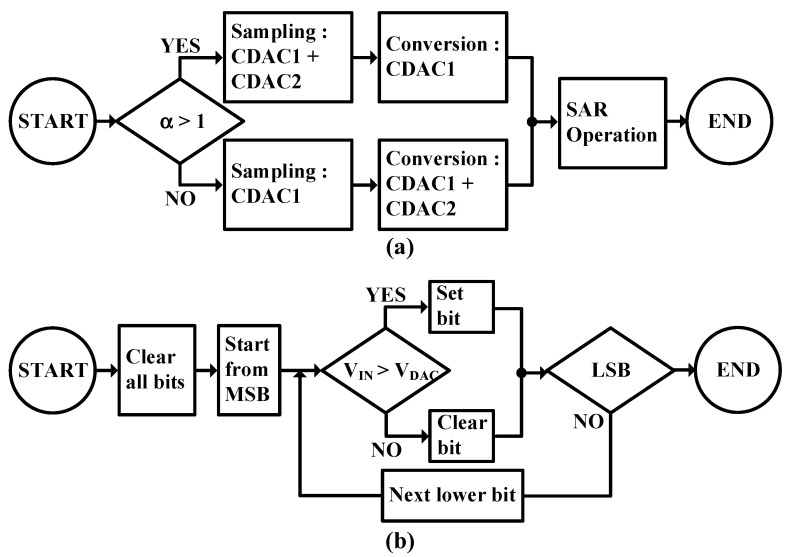
Flow charts of (**a**) SAR-based quantizer and (**b**) SAR logic.

Figure 13 illustrates the complete operation of the SAR-based quantizer through two interconnected flow charts. The first flow chart (a) demonstrates the high-level quantizer operation with feedback gain control, while the second flow chart (b) details the SAR logic implementation.

In flow chart (a), the operation begins by evaluating the feedback gain α. When α is greater than 1, the system enters a path where CDAC1 + CDAC2 is used during the sampling phase, followed by CDAC1 during the conversion phase. Conversely, when α is less than or equal to 1, the system takes an alternate path where CDAC1 alone is used for sampling, followed by CDAC1 + CDAC2 for conversion. Both paths ultimately converge to the SAR operation before completion, ensuring proper quantization regardless of the feedback gain configuration. Flow chart (b) details the SAR logic implementation, beginning with an initialization phase where all bits are cleared. The conversion process starts from the Most Significant Bit (MSB) and proceeds through a systematic comparison loop. During each iteration, the input voltage (V_IN_) is compared with the DAC output voltage (V_DAC_). If V_IN_ exceeds V_DAC_, the current bit is set; otherwise, it is cleared. This process continues with each successive bit until the Least Significant Bit (LSB) is reached. After processing the LSB, the conversion is complete, and the system signals the end of operation.

This dual flow chart structure effectively captures both the gain-dependent operation of the quantizer and the bit-by-bit successive approximation process, providing a comprehensive visualization of the complete quantization sequence. The design demonstrates how the non-unity feedback gain implementation is seamlessly integrated with traditional SAR operation, resulting in an efficient and versatile quantization system.

## 6. Experimental Results

**Figure 14:**
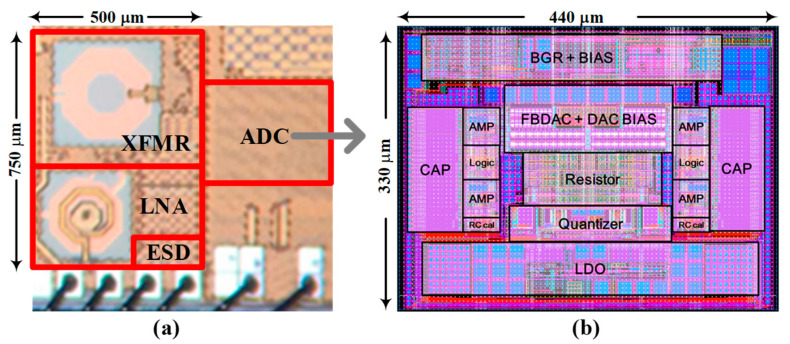
(**a**) Chip microphotograph and (**b**) layout view of QBP-CTΣΔM ADC.

The proposed receiver was fabricated in a 28 nm CMOS process, as shown in Figure 14a. The chip layout features an RF front-end area of 0.375 mm^2^ and a QBP-CTΣΔM ADC area of 0.145 mm^2^. In BLE-mode operation, the receiver achieves a sensitivity of −95 dBm at a 10⁻^3^-bit error rate while consuming 4.08 mW from a 1.0 V supply. Figure 14b shows the layout view of the QBP-CTΣΔM ADC that draws 0.81 mW including power consumption from the bias circuit for current distribution to active blocks, input/output drivers, and clock distribution circuitry.

**Figure 15:**
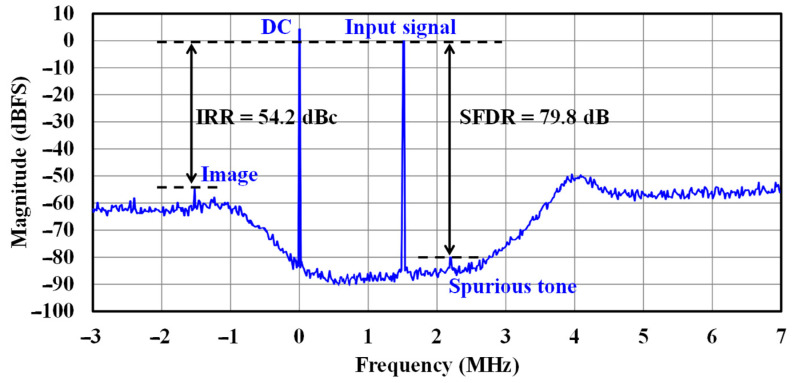
Output spectrum of the RX output with a low-IF signal at 1.5 MHz.

SNR measurements were conducted using a peak low-IF signal at 1.5 MHz with a 100 Hz resolution, as illustrated in Figure 15. The feedforward architecture results in minimal harmonic distortion at the output. The design achieves a peak SFDR of 79.8 dBc, with an impressive image rejection ratio of 54.2 dBc during measurements.

**Figure 16:**
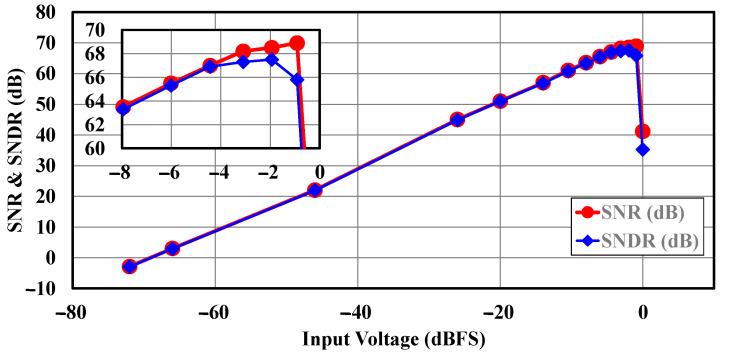
Measured SNR and SNDR for various input voltages.

The measured SNDR and SNR across different input signal levels are plotted in Figure 16. The ADC demonstrates peak performance with an SNDR of 67.5 dB at −2 dB relative to full scale (dBFS) and a peak SNR of 68.9 dB at −1 dBFS. For reference, the full-scale signal (0 dBFS) corresponds to a differential peak-to-peak voltage (V_P-P_) of 1 V. The ADC achieves a dynamic range of 70.3 dB across its 2 MHz signal BW.

**Figure 17:**
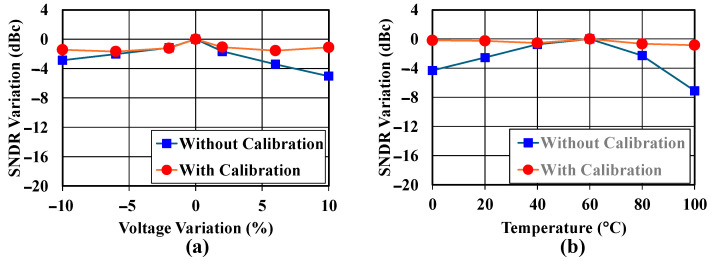
Measured SNDR with (**a**) voltage and (**b**) temperature variations.

Figure 17 shows the performance stability of the ADC’s SNDR under varying operating conditions. The voltage variation demonstrates that the SNDR responds to supply voltage changes from −10% to +10%. Without RC calibration, the SNDR significantly degrades by −16 dBc at 10% voltage variation. With calibration enabled, the variation is contained within ±2 dBc. The temperature variation shows SNDR performance from 0 °C to 100 °C. The uncalibrated system’s SNDR degrades by −8 dBc at 100 °C, while the calibrated system maintains performance within ±2 dBc across the temperature range. These results validate the effectiveness of the RC calibration scheme in maintaining stable ADC performance across both voltage and temperature variations, which is essential for reliable operation in IoT applications.

**Figure 18:**
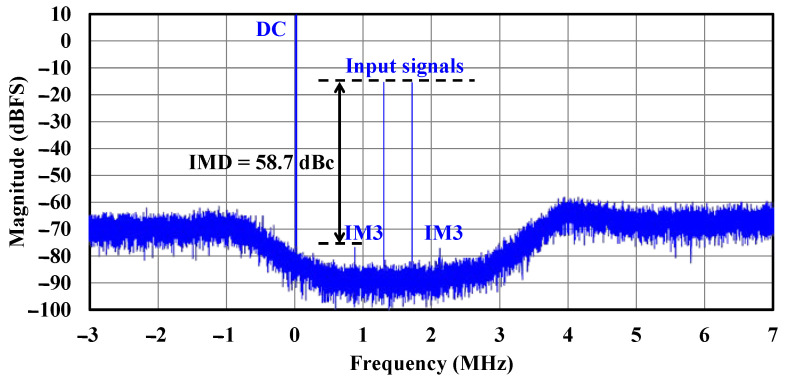
Measured intermodulation performance.

Intermodulation testing was performed to characterize the QBP-CTΣΔM ADC’s linearity. When presented with two strong input signals at −15 dBFS at frequencies of 1.3 GHz and 1.7 GHz, the resultant intermodulation products measure 58.7 dBc below the fundamental tones, as shown in Figure 18.

Table 1 presents a performance comparison between the proposed receiver and previously reported IoT receivers with ΣΔM [35,36,37,38,39,40,41]. Our design implements a low-IF architecture for BLE standards with an intermediate frequency (IF) of 1.5 MHz. The CT-QΣΔM ADC achieves an effective number of bits (ENOB) of 10.9, operates at a sampling frequency of 32 MHz, and provides a BW of 2 MHz. To enable fair comparison, we evaluate the ADC performance using two established figures of merit: FOM_S_ [42] and FOM_W_ [43]. The FOM_S_ metric emphasizes resolution and dynamic range improvements, with higher values indicating better performance. Conversely, FOM_W_ focuses on energy efficiency, where lower values represent better performance. The proposed CT-QBP ΔΣM ADC achieves an FOM_S_ of 164.3 dB. While this figure is lower than some IoT sensor applications [35,36,40,41], due to our higher intermediate frequencies, our design demonstrates superior energy efficiency with the best FOM_W_ among state-of-the-art implementations [35,36,37,38,39,40,41].

## 7. Conclusions

This paper presented a low-power CMOS receiver with a complex continuous-time delta–sigma ADC optimized for IoT applications in the 2.4 GHz band. The proposed design incorporates a quadrature bandpass continuous-time delta–sigma ADC that achieves an ENOB of 10.9 bits while consuming only 0.81 mW from a 1.0 V supply. The ADC successfully provides a BW of 2 MHz while operating at a 32 MHz sampling frequency, making it ideally suited for BLE applications.

The receiver architecture demonstrates several significant performance achievements through its innovative features. The carefully optimized low-IF architecture operating at 1.5 MHz IF delivers strong image rejection capability with a measured IRR of 54.2 dBc. The design also achieves an excellent SFDR of 79.8 dBc, demonstrating its robust signal-handling capabilities. Most notably, the architecture achieves superior energy efficiency with an FOM_W_ of 103.2 fJ/conv, representing the best figure among current state-of-the-art implementations.

The complete receiver system demonstrates an impressive sensitivity of −95 dBm at a 10⁻^3^-bit error rate while maintaining low power consumption of 4.08 mW, with the ADC portion requiring only 0.81 mW. The design implementation in 28 nm CMOS technology achieves a compact form factor, occupying just 0.375 mm^2^ for the RF front-end and 0.145 mm^2^ for the ADC. These specifications demonstrate that the proposed architecture successfully addresses the demanding requirements of modern IoT applications, particularly for BLE standards, by delivering an optimal balance between power efficiency, performance, and integration density.

The achieved specifications make this design particularly well suited for battery-powered IoT sensor nodes where both power efficiency and reliable wireless connectivity are critical requirements. The demonstrated architecture provides a robust foundation for advancing the development of next-generation low-power wireless sensor networks.

## Figures and Tables

**Table 1:** Performance summary of the proposed RX and comparisons to the state of the art with ΣΔM.

	This Work	[35] MEJ24	[36] CICC21	[37] TMTT20	[38] Sensors17	[39] JSSC18	[40] TMTT17	[41] TVLSI17
Technology (nm)	28	180	180	28	110	160	180	65
System	BLE	IoT Sensor	IoT Sensor	BLE	BLE	IoT Sensor	GNSS	BLE
RX Architecture	Low-IF	N/A	N/A	Low-IF	Low-IF	N/A	Low-IF	Low-IF
ADC Type	CT-QΣΔM	DT-ΣΔM	DT-ΣΔM	CT-ΣΔM	CT-ΣΔM	SAR + DT-ΣΔM	CT-QΣΔM	CT-QΣΔM
ADC ENOB (bit)	10.9	16.1	14.5	10.5	N/A	19.3	10.0	10.2
Sampling Frequency (MHz)	32	2.5	0.2	32	128	2	460	200
Signal BW (MHz)	2	0.01	0.0008	2	1	0.001	33	5
IRR (dBc)	54.2	N/A	N/A	32	N/A	N/A	59.3	59.6
DR (dB)	70.3	102.6	94.1	66	N/A	120.3	64	74.3
SNDR (dB)	67.5	98.6	89.3	65.2	N/A	118.1	62.1	62.9
SNR (dB)	68.9	101.5	91.9	N/A	N/A	119.1	64.5	64.4
Supply Voltage (V)	1.0	1.8	1.5	1.04	N/A	1.8	1.8	1.2
ADC Power (mW)	0.81	1.3	0.004	* 1.2	2.4	0.28	* 25.4	4.2
ADC Area (mm^2^)	0.145	0.648	0.75	0.18	0.24	0.25	0.73	0.39
** FOM_S_ (dB)	164.3	171.5	177.1	158.2	N/A	185.8	155.1	165.1
** FOM_W_ (fJ/conv.)	103.2	934.2	104.8	201.7	N/A	213.1	369.8	368.1

* Estimation by subtracting other circuit power consumption from the total receiver power. ** ${\mathrm{FOM}}_{S}=\mathrm{SNDR}\left(\mathrm{dB}\right)+10\cdot \log_{10}\left[\frac{\mathrm{BW}(\mathrm{Hz})}{P(W)}\right]$, ${\mathrm{FOM}}_{W}=\frac{P(W)}{2\cdot \mathrm{BW}(\mathrm{Hz})\cdot 2^{\mathrm{ENOB}}}\;\mathrm{where}\;\mathrm{ENOB}=\frac{\left[\mathrm{SNDR}\left(\mathrm{dB}\right)-1.76\right]}{6.02}$.

## Data Availability

Data are contained within the article.
